# Adaptation response to pitch shift during speech is attenuated upon relearning

**DOI:** 10.3389/fnhum.2025.1689023

**Published:** 2026-01-07

**Authors:** Jessica L. Gaines, Corby L. Dale, John F. Houde, Srikantan S. Nagarajan

**Affiliations:** 1 UC Berkeley - UCSF Graduate Program in Bioengineering, San Francisco, CA, United States; 2 Department of Radiology, University of California, San Francisco, San Francisco, CA, United States; 3 Department of Otolaryngology – Head and Neck Surgery, University of California, San Francisco, San Francisco, CA, United States

**Keywords:** speech motor control, sensorimotor adaptation, motor learning, fundamental frequency, pitch

## Abstract

**Introduction:**

During sensorimotor adaptation, participants respond to a persistent sensory error by shifting behavior to oppose the error. This phenomenon has been measured in multiple motor tasks in which sensory feedback is experimentally altered to artificially introduce an error. Tasks involving multiple cycles of altered and unaltered feedback have been used in the arm reaching domain to understand the mechanisms of un-learning and re-learning a response to an error, but re-learning within a single session has not been studied in the domain of fundamental frequency (f0) control during speech.

**Methods:**

In this study, participants responded to three alternating blocks of f0-shifted and unshifted auditory feedback during a single-word speech task.

**Results:**

It was found that on average, adaptation magnitude decreased in the second and third blocks of shifted feedback compared to the first. This illustrates an attenuation effect similar to that observed in studies of implicit learning in arm reaching tasks.

**Discussion:**

These results support the understanding of f0 control as an implicit learning phenomenon and help place f0 control in the context of motor control in general.

## 1 Introduction

Sensorimotor adaptation is a phenomenon that occurs in response to a persistent sensory error during movement. When an error is encountered repeatedly, individuals tend to counteract the error by shifting their behavior in the opposite direction of the error on subsequent movements. This phenomenon has been observed in many movement tasks including speech (Jones and Munhall, 2000; Houde and Jordan, 2002) and arm movement (Krakauer et al., 1999). In speech adaptation tasks, participants listen to the sound of their voice through headphones in near-real time while producing speech. Experimenters introduce an artificial error by modifying components of the speech sound such as the fundamental frequency (f0), which is perceived as vocal pitch, or formant frequencies (F1, F2), which are involved in categorization of vowel sounds (Jones and Munhall, 2000; Houde and Jordan, 2002). In arm movement visuomotor rotation tasks, participants reach toward a visual target while the view of their arm is obscured by a digital screen with a moving cursor to indicate hand position. Experimenters introduce a perceived error by altering the movement of the cursor from the true direction of the reach by some angle (Krakauer et al., 1999). Understanding how the brain adapts to these types of errors can provide insights related to neuro-motor control, the mechanisms by which the brain controls muscles to achieve a movement goal.

Sensorimotor learning is often categorized into two types: implicit learning and explicit learning. Explicit learning has been defined in many studies as a conscious, cognitive strategy, while implicit learning is automatic and beyond conscious control (Mazzoni and Krakauer, 2006; Krakauer, 2009; Lametti et al., 2020). However, the definitions of explicit and implicit learning vary in motor control literature. (Taylor et al. 2014) framed explicit learning as a behavioral change in response to a target error (the difference between the intended position and the true position), while implicit learning is a response to sensory prediction error (the difference between the position estimated by the neuro-motor system's internal models of the body and the true position in space). (Morehead et al. 2015) expanded this definition to argue that explicit learning is the adaptation of an action selection process, while implicit learning is the adaptation of a movement execution process, and specifically excluded awareness of the error from their definitions. (Tsay et al. 2024) proposed that explicit strategy may include some automatic components when a control policy is well learned.

Using the first definition, several studies have supported the idea that speech involves only implicit learning. In the domain of formant adaptation, participants have been found to be unable to articulate any intention to change their speech behavior (Kim and Max, 2021) and unable to ignore altered feedback even when instructed to do so (Munhall et al., 2009). Additionally, added cognitive load in the form of simultaneous adaptation in F1 and visuomotor domains did not affect the magnitude of the F1 adaptation (Lametti et al., 2020), further suggesting the automatic nature of F1 adaptation. The domain of f0 control, however, may be less clear. It has been shown that participants, even trained singers, are unable to ignore alterations in f0 (Keough et al., 2013). However, a dual task condition did affect the magnitude of f0 adaptation during a singing task (Scheerer et al., 2016b), suggesting that f0 adaptation may not be entirely independent of cognition. Furthermore, not all definitions of explicit and implicit learning depend on conscious strategy. The inability of speakers to ignore altered feedback may not necessarily indicate properties of learning that are relevant to the development of computational models of speech motor control, such as whether learning is a response to a target error or a prediction error. The characterization of speech motor learning as primarily implicit may therefore need continued investigation.

Studies in the arm reaching domain have isolated implicit and explicit learning without relying on intentionality and found that behavior differs between the two processes in response to re-learning, in which participants encounter a second phase of altered feedback after the first, separated by some rest or washout phase in which no alteration is applied. For example, (Avraham et al. 2021) isolated implicit learning by sometimes removing visual feedback or by fixing it to a constant value, thus removing the role of target error. It was observed that participant responses to a repeated visuomotor adaptation task differed for each type of learning. When target error was present (explicit learning conditions), participants exhibited savings, adapting at a faster rate in the second repetition of the task. When meaningful target error was not available (implicit learning conditions), participants instead exhibited attenuation, adapting at a slightly slower rate and with smaller magnitude in the second repetition of the task. This attenuation of adaptation under implicit learning conditions has been supported by other recent studies in the arm reaching domain (Hamel et al., 2021; Leow et al., 2020).

How these findings may be extensible to f0 control in speech has not been explicitly explored. Several related studies have performed multiple adaptations in vocal tasks while investigating other research questions, but none have focused on repeated adaptation or measured repeated cycles of f0 adaptation in a speech task in a single session (Alemi et al., 2020; Kapsner-Smith et al., 2024; Kim et al., 2025; Hawco and Jones, 2010). Thus we conducted a study of identical cycles of f0 adaptation in a single session to facilitate the comparison between limb and speech motor control and provide a better understanding of the extent to which neural mechanisms of control are similar and different between the two domains. Perceived f0 was shifted up or down in three altered-feedback blocks, each separated by a lengthy washout block during which feedback was unaltered. Because speech motor learning is currently understood to be primarily implicit, we hypothesized that the magnitude of f0 adaptation would be slightly attenuated after the first cycle, in alignment with implicit re-learning studies in the visuomotor domain.

## 2 Materials and methods

### 2.1 Participants

Twenty-two speakers were recruited to participate in the study. Two participants were excluded from analysis due to extremely high utterance-to-utterance variance in f0 (more than 2 standard deviations greater than the mean variance of all participants). The remaining 20 participants (11 female) had mean age of 40.9 ± 14.9 (standard deviation). All future references to the group of participants is intended to refer only to the 20 participants included in analysis, unless otherwise specified. Participants reported no history of neurological or speaking disorders and took a hearing test. Six participants over 49 years of age had a hearing threshold >20dB at high frequencies (3 participants at 2,000, 4,000, and 8,000 Hz, 1 at 4,000 and 8,000 Hz, and 2 at 8,000 Hz only). One additional participant over 49 years of age tested >20dB at 250, 500, and 8,000 Hz. One participant under 49 years of age tested >20dB at 2,000 Hz only. All remaining participants passed a hearing test at 20dB for frequencies between 250 Hz and 8,000 Hz. All participants were native speakers of English, with additional fluent language background in Spanish (4 participants), Urdu (1 participant), and Arabic (1 participant). Research activities were approved by the Institutional Review Board of the University of California, San Francisco.

### 2.2 Experiment setup

Stimulus presentation and word production took place in a sound-attenuated booth (Model C-14A LP Mod. Rev., Eckel Noise Control Technologies, Morrisburg, ON, Canada). Participants spoke into a head-mounted microphone (Headset microphone C520, AKG Acoustics, Los Angeles, CA, USA) placed at 3cm from the corner of the mouth. Speech sounds were processed by a microphone preamplifier (HR-MP2, Radio Design Labs, Prescott, AZ, USA), and then by the sound-modifying software Feedback Utility for Speech Production (FUSP), which (on altered trials) shifted the fundamental frequency of the sound and played the altered or unaltered sound in near-real time though over-ear headphones.

Speaking prompts were presented on a computer screen. The prompt word was “odd” (/ad/). The initial vowel sound /a/ was chosen to align with previous pitch adaptation studies (e.g., Burnett et al., 1998; Jones and Munhall, 2000; Scheerer et al., 2016a). Ten percent of trials, randomly interleaved with the “odd” trials, gave the prompt word “eat”. These trials were intended to keep the participants alert to the task and were discarded during analysis. One participant was prompted with “eat” in 15% of trials.

### 2.3 Calibration of sound level for feedback presentation

The loudness of the feedback was calibrated as follows. A sound source (Gigaworks T20 Series II speaker, Creative Labs, Singapore) was adjusted to a sound level of 75dB as measured 10 cm away by a sound level meter (Type 2260, Brüel & Kjær, Inc., Nærum, Denmark) with a free-field microphone (Type 2669 microphone preamplifier and Type 4189 1/2” prepolarized free-field microphone, Brüel & Kjær, Inc., Nærum, Denmark). This distance approximates the distance between the mouth and the ear. The loudness of the feedback was calibrated such that this sound level was presented at 80 dB through headphones as measured by the sound level meter and an artificial ear (Type 4152, Brüel & Kjær, Inc., Nærum, Denmark) when the headset microphone was placed 3 cm from the source (resting distance of the head-mounted microphone). Thus the feedback was presented at +5 dB louder than the participants would hear their air-conducted voice (as recommended by Weerathunge et al., 2020). Participants were asked before the start of the experiment if the loudness was uncomfortable. Two participants requested a reduction in loudness to +4dB louder than their air-conducted voice, and one requested a reduction to +3dB.

### 2.4 Instructions

Before the start of the task, participants were instructed to read the words aloud at their normal speaking rate and volume. They were asked to speak clearly and consistently, that is, to say the word the same way each time. Participants were notified during recruitment that their feedback may be altered, however, the nature of the alteration was not specified and they were not reminded of possible feedback alteration during the task instructions. Participants were asked at the end of the session whether they noticed anything unique about the feedback presented to them. Three of the included participants were able to identify that their vocal pitch had been changed. Both of the participants excluded for extremely high variability identified that their pitch had been changed.

### 2.5 Trials

**Figure 1:**
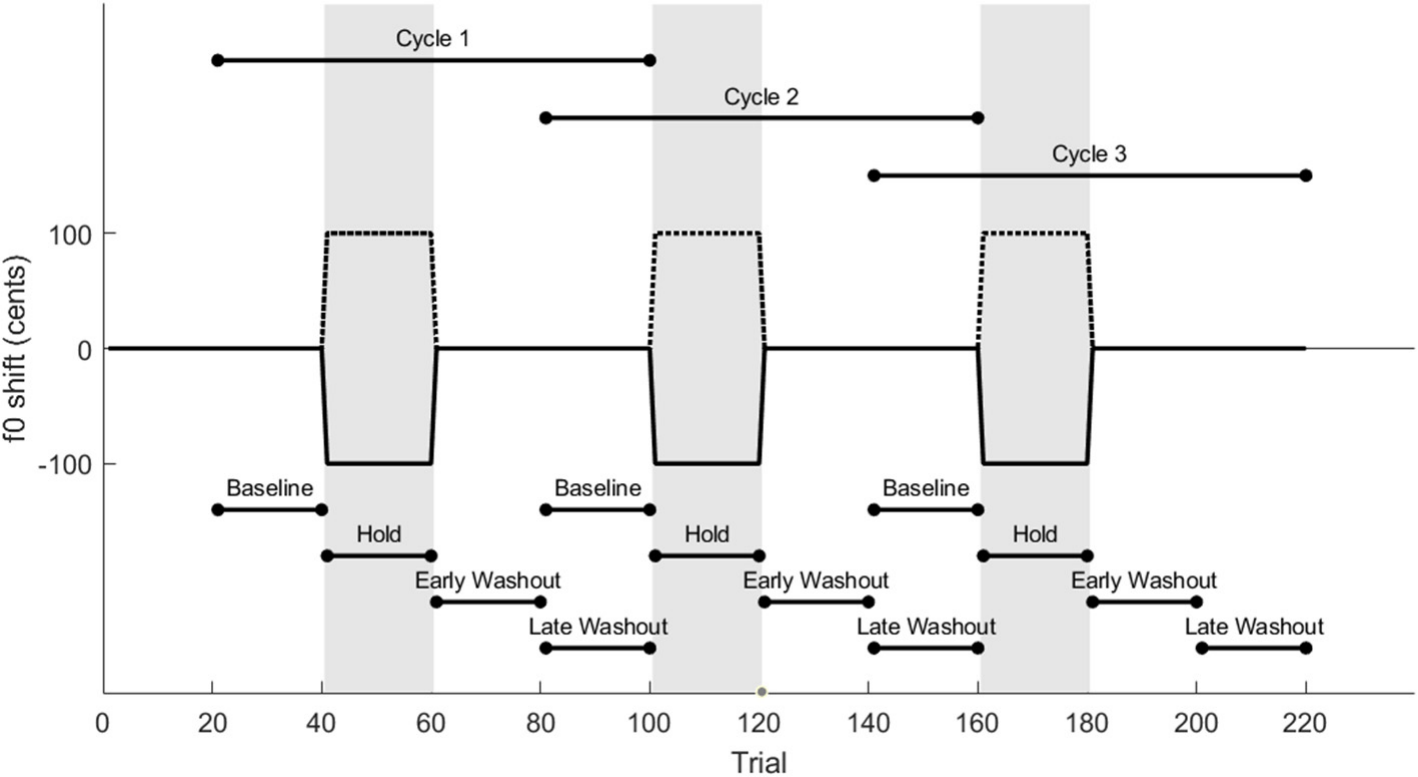
Phases of alteration and analysis. Participants received alternating blocks of 20 auditory feedback-altered and 40 unaltered trials for a total of 220 trials and 3 altered blocks. The auditory feedback alteration was a 100-cent upshift or downshift of f0. Periods of trials used for analysis are annotated for each phase and each cycle.

A total of 220 trials were presented to each participant. A baseline period of 40 unaltered trials was followed by three repetitions of 20 f0-shifted trials (hold phase) and 40 unaltered trials (washout phase) as seen in Figure 1. A hold-phase length of 20 trials was selected based on previous pitch adaptation studies showing that response magnitude begins to asymptote within 5–10 trials (Scheerer et al., 2016a; Keough et al., 2013; Alemi et al., 2020). The washout phase was selected to be twice the length of the hold phase. During hold phases, f0 was shifted up or down by 100 cents for the whole trial, starting at voice onset. This was perceived as a slightly higher or lower vocal pitch than was actually produced. The direction of the shift was randomly counterbalanced across participants but consistent within each participant, that is, each participant received either three blocks of upshifted f0 or three blocks of downshifted f0. The outlier participants who were removed from analysis both received downshifted feedback, so among the included participants, 11 received upshifted f0 and 9 received downshifted f0.

During each trial, the prompt appeared on the screen for 1.7 s, followed by a pause of 1.7 s plus a 0–1 second jitter before the next trial. Trials proceeded automatically except that every 20th trial, participant input (pressing the space bar) was required to proceed.

### 2.6 Data preprocessing

The raw audio signal from each utterance was preprocessed in MATLAB (MathWorks, Inc., Natick, MA, USA) using WaveViewer (https://github.com/SpeechNeuroscienceLab/Wave-Viewer). After “eat” trials were discarded, 1.5% of remaining trials were discarded for non-speech or poor pitch tracking in the time period between 50 and 150 ms after voice onset. The remaining “good” trials were retained for analysis. Each good trial was manually marked at voice onset and at the end of the vowel sound or the end of stable pitch tracking, whichever came first. The median f0 from 50 to 150 ms after voice onset was extracted for each trial. This time period excluded the first 50 ms during which pitch tracking was often poor and the time after 150 ms, when online feedback control may start to affect behavior (Burnett et al., 1998).

### 2.7 Analysis

Next, the median f0 extracted from each trial was normalized to the baseline f0 of each participant for each cycle. Each cycle (consisting of one repetition of baseline phase, hold phase, and a washout phase split into early and late periods) was normalized separately in order to reduce the effects of any drift in pitch over many trials and provide an equivalent comparison between each of the three cycles. The mean across the final 20 trials of each baseline phase was used for normalization. The first 20 trials of each baseline phase were not used for normalization to minimize the impact of any after-effects from a previous hold phase or, in the case of the first cycle, changes in pitch as participants became accustomed to hearing their voice through headphones. The f0 for each trial was thus normalized to cents using Equation 1, where f_baseline_ is the mean of the 20 trials preceding each hold phase. In order to pool across participants who received upshifted and downshifted feedback, the normalized f0 values of participants who received upshifted feedback were sign-inverted (reflected across y = 0) so that positive cents indicated a behavioral change in f0 opposite the direction of the perceived shift.


$$\begin{array}{l} {\text{f}}_{\text{cents}}=1200*{\text{log}}_{2}\left(\frac{{\text{f}}_{\text{hertz}}}{{\text{f}}_{\text{baseline}}}\right) \end{array}$$ (1)

### 2.8 Statistical analysis

To test the main hypotheses that vocal pitch adaptation occurs during feedback perturbation (the hold phase), and that this effect is attenuated across multiple presentations (cycles) in a session, a linear mixed-effects model (fitlme, Matlab vR2024b, Natick, MA) was used to determine significant differences in adaptation across phase of the experiment (baseline, hold, early washout, late washout), presentation cycle (first, second, or third), and shift direction (downshift or upshift) where “baseline”, “first”, and “downshift” were used as reference categories. All two-way and three-way interaction terms between phase, cycle, and shift direction and a random effect of participant were also included. The model was therefore as follows:


$$\begin{array}{r} \text{f}0\sim 1+\text{phase}+\text{cycle}+\text{shift}+\left(\text{phase*cycle}\right)+\left(\text{cycle*shift}\right)\\ +\;\left(\text{phase*shift}\right)+\left(\text{phase*cycle*shift}\right)+\left(1|\text{participant}\right) \end{array}$$ (2)

Each phase submitted to the model consisted of 20 trials; the baseline phase was defined as the twenty trials immediately before each hold phase. The data from the 4,200 single-trial observations were fit to this model (20 trials x 4 phases x 3 cycles x 20 subjects = 4,800, remove 600 “eat” trials and bad trials). For all results, significant effects are reported at a threshold of *p* < 0.05. Effect sizes are derived from the model as fixed effects coefficients (the model intercept for reference categories, or the category coefficient summed with the model intercept for all other categories). The significance of categorical variables and their interactions across all levels were determined using anova marginal tests (anova, Matlab vR2024b, Natick, MA). A significant interaction obtained in the main model was examined in posthoc tests for phase and cycle. Posthoc tests were conducted by fitting linear mixed-effects models to subsets of the data from one cycle (f0~1+phase+(1|participant)) or phase (f0~1+cycle+(1|participant)) at a time. Again, “baseline” was used as the reference category for phase and “first” was used as the reference category for cycle.

Additional second-level analyses were conducted using Pearson's Correlation Coefficients to examine within-subject relationships across cycles (corr, Matlab vR2024b, Natick, MA), and Fisher's exact tests of independence to reveal potential patterns in subject behavior (fishertest, Matlab vR2024b, Natick, MA). A cluster-based permutation test (permutest, https://www.mathworks.com/matlabcentral/fileexchange/71737-permutest; Maris and Oosterveld, 2007) was used to determine significant clusters of trials in trial-by-trial data.

## 3 Results

### 3.1 Multiple adaptation

ANOVA marginal tests on the full linear mixed effects model revealed a significant main effect of phase (F_[3, 4176]_ = 3.19, *p* = 0.02) and a significant interaction between phase and cycle (F_[6, 4176]_ = 2.91, *p* = 0.007). The main effects of cycle and shift, and all other interaction terms were not significant. Fixed effects coefficients from the full model showed a significant main effect of hold phase (t_[4176]_ = 3.07, *p* = 0.002) with an effect size of 21.2 cents (β = 21.17, SE = 6.89). The main effects of early washout and late washout were not statistically significant (t_[4176]_ = 1.29, *p* = 0.2; t_[4176]_ = 1.25, *p* = 0.2). The main effects of the second and third cycle were not significant (t_[4176]_ = -0.05, *p* = 0.96; t_[4176]_ = −0.03, *p* = 0.97), but a significant interaction was found between late washout phase and third cycle (t_[4176]_ = −3.18, *p* = 0.001).

**Figure 2:**
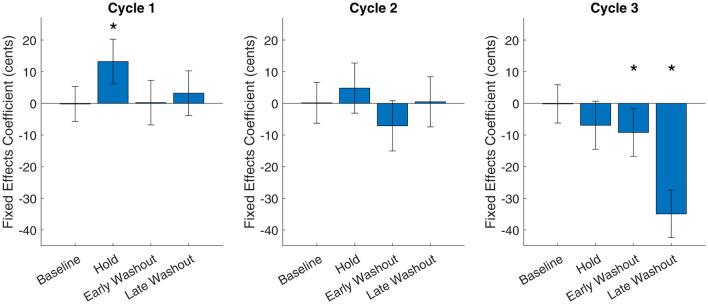
Fixed effects coefficients from posthoc tests by cycle. For each cycle, the fixed effects coefficient of the model intercept is shown for baseline phase. The fixed effects coefficients of the other phases are intercept-adjusted for direct comparison to zero. Error bars indicate standard error from the linear mixed effects model. For intercept-adjusted bars, standard error is combined with that of the intercept. Statistically significant phases (different from zero for baseline, different from baseline for all other phases) at significance level *p* < 0.05 are marked with an asterisk (*).

Posthoc tests were used to investigate the significant interaction between phase and cycle. Since shift direction was not significant in the full model, it was removed from the posthoc models. In the first cycle, hold phase was significantly different from baseline (t_[1421]_ = 3.05, *p* = 0.002) with an effect size of 13.2 cents (β = 13.40, SE = 4.39; intercept = -0.19, SE = 5.52) while early and late washout phase were not significant (early washout: t_[1421]_ = 0.10, *p* = 0.92; late washout: t_[1421]_ = 0.77, *p* = 0.44). In the second cycle, no significant difference from baseline was found (hold phase: t_[1394]_ = 1.01, *p* = 0.31; early washout: t_[1394]_ = −1.58, *p* = 0.11; late washout: t_[1394]_ = 0.07, *p* = 0.94). In the third cycle, hold phase was not significantly different from the baseline reference category (t_[1373]_ = -1.47, *p* = 0.14) but early and late washout phases were significant (early washout: t_[1373]_ = -2.03, *p* = 0.04; late washout: t_[1373]_ = −7.76, *p* < 0.001) due to unexpected behavior in the final trials of the experiment. Posthocs by cycle are visualized in Figure 2.

**Figure 3:**
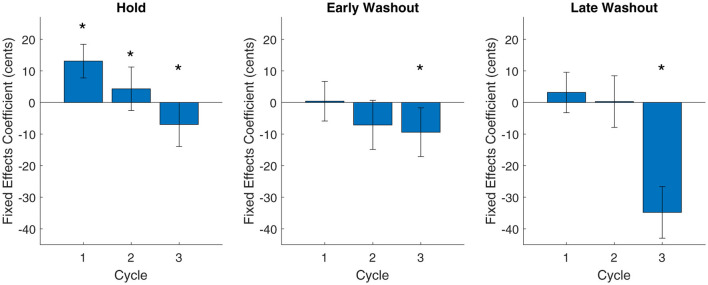
Fixed effects coefficients from posthoc tests by phase. For each phase, the fixed effects coefficient of the model intercept is shown for cycle 1. The fixed effects coefficients of the other cycles are intercept-adjusted for direct comparison to zero. Error bars indicate standard error from the linear mixed effects model. For intercept-adjusted bars, standard error is combined with that of the intercept. Statistically significant cycles (different from zero for cycle 1, different from cycle 1 for all other cycles) at significance level *p* < 0.05 are marked with an asterisk (*).

In the baseline phase, no differences between cycles were significant (cycle 2: t_[1059]_ = 0.04, *p* = 0.97; cycle 3: t_[1059]_ = 0.01, *p* = 0.99). In the hold phase, the first cycle was significantly different from zero with effect size of 13.1 cents (t_[1019]_ = 2.46, *p* = 0.01, intercept = 13.10, SE = 5.32). The second and third cycles were significantly different from the first cycle with effect sizes of 4.3 and −7.0 cents, respectively, (or −8.8 and −20.1 cents lower than the first cycle; cycle 2: t_[1019]_ = −2.00, *p* = 0.046, β = −8.78, SE = 4.39; cycle 3: t_[1019]_ = −4.45, *p* < 0.001, β = −20.07, SE = 4.51) indicating a significant attenuation of adaptation. In the early washout phase and late washout phase, only the third cycle was significant (early washout, cycle 3: t_[1061]_ = −2.16, *p* = 0.03; late washout, cycle 3: t_[1049]_ = −7.45, *p* < 0.001), again reflecting the unexpected behavior in the final trials of the experiment. Posthocs by phase are visualized in Figure 3.

### 3.2 Adaptation timecourse

**Figure 4:**
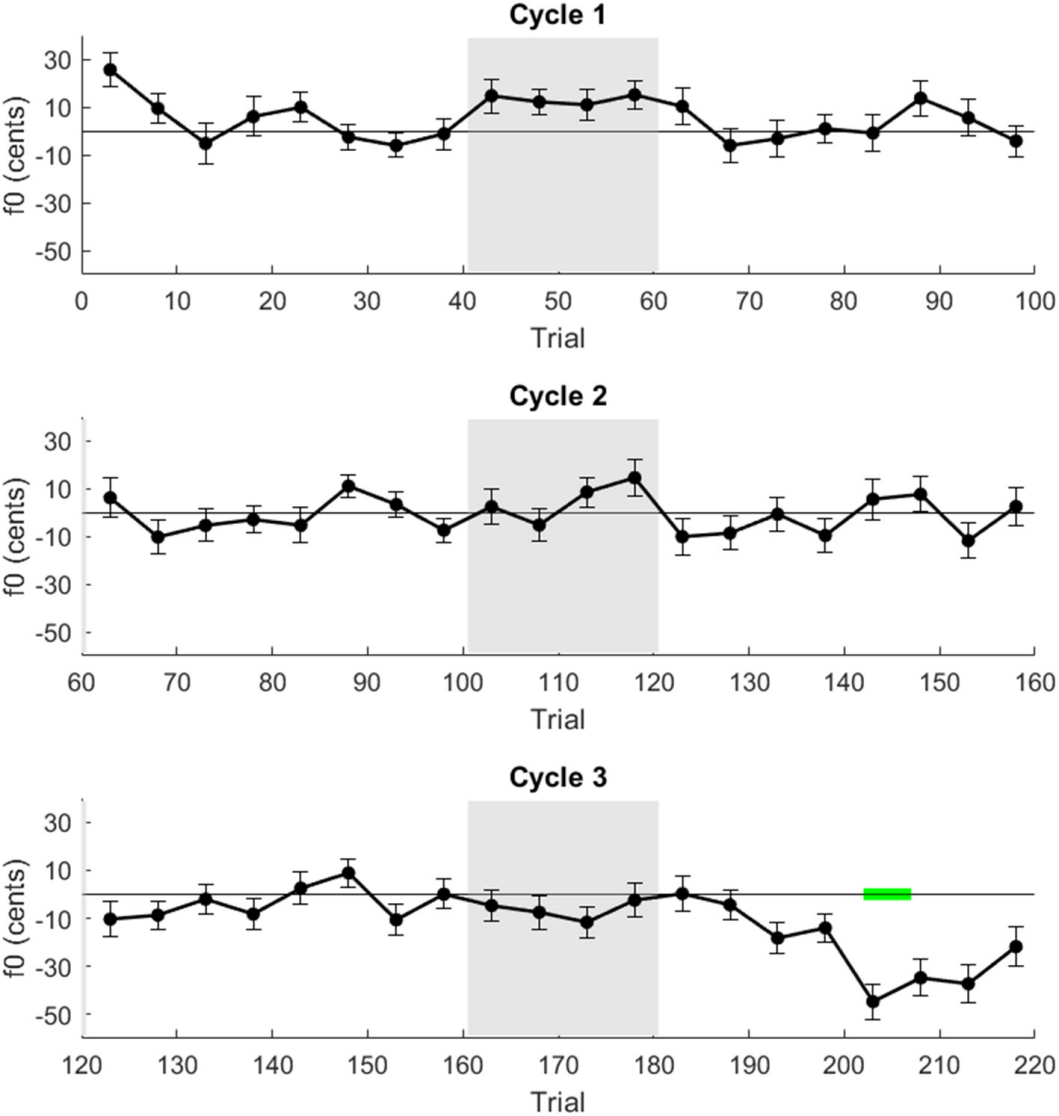
Timecourse of adaptation. Average f0 across speakers in five-trial blocks. Error bars indicate standard error. Gray shading indicates altered auditory feedback and green highlight indicates significant clusters by a cluster permutation test.

The progression of adaptation over the course of the experiment is shown in Figure 4. Although adaptation is visible in the first cycle of altered feedback, any adaptation response is difficult to distinguish from noise in the second and third cycles, echoing the results of the linear mixed-effects model analysis. A cluster-based permutation test did not identify any significant clusters during the hold phases, despite the significance of the first hold phase in the linear mixed effects model when directly compared to the previous baseline phase as reference.

### 3.3 Individual variability in adaptation behavior

**Figure 5:**
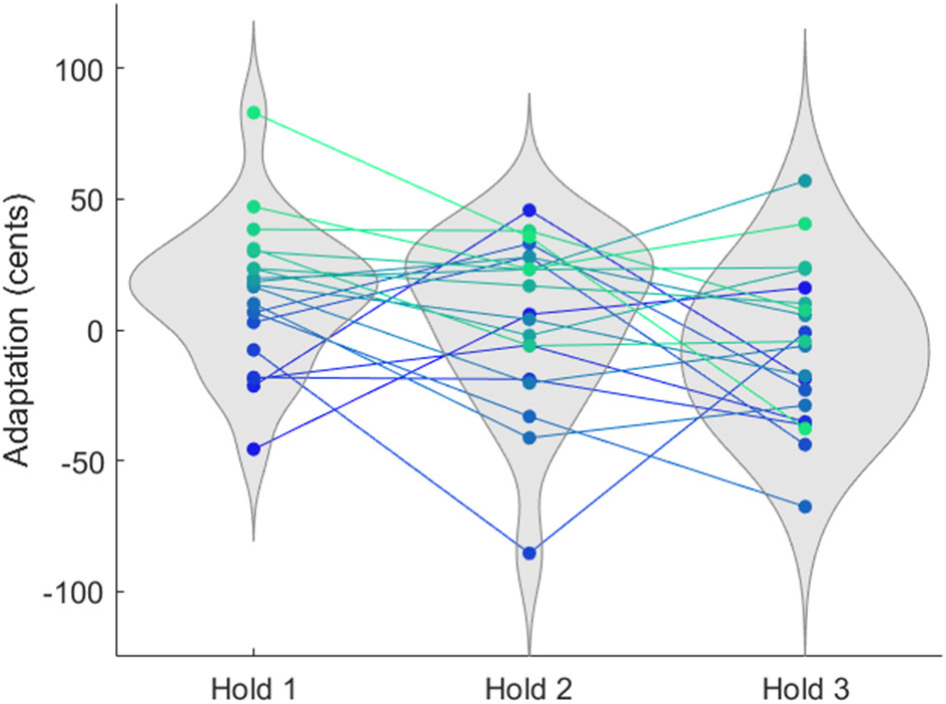
Adaptation magnitude of individual participants. The adaptation magnitude, as measured by the mean f0 of the trials of each hold phase, is shown for each participant in each hold phase. Individual participants are represented by markers of a single color across cycles. Colors are in a spectrum from green to blue indicating adaptation magnitude in the first hold phase. Participants show large individual variability in their behavioral response to feedback alteration, including some whose behavior followed the same direction as the alteration. There was also large individual variability in adaptation between hold phases, with participants showing decreased, increased, and similar magnitudes across hold phases.

On average across all participants, adaptation magnitude attenuated significantly after the first presentation cycle. To understand the role of individual participants in this group effect, the trajectory of each participant's adaptation magnitude was plotted across cycles. Participants varied widely in their adaptation magnitude for each cycle, and in change in adaptation magnitude across cycles (Figure 5).

Each cycle had participants who shifted their produced pitch in the opposite direction of the perturbation (positive adaptation) and participants who shifted their produced pitch in the same direction as the perturbation (negative adaptation; cycle 1: 25% of participants; cycle 2: 40% of participants, cycle 3: 60% of participants). These were often not the same participants, with only 30% of participants showing positive adaptation across all cycles and 15% participants showing negative adaptation across all cycles, while the remaining 55% of participants showed positive adaptation during some hold phases and negative adaptation during others. A Fisher's exact test showed no significant difference in the distribution of positive and negative adapters across cycles.

Previous work has shown poor test-retest reliability in f0 adaptation across sessions spaced weeks apart (Kapsner-Smith et al., 2024), but this effect has not been previously explored across cycles in a single session. Consistent with this previous study, participants in the current study were highly variable in their behavior between cycles. Although as a group, adaptation magnitude decreased after the first cycle, this was not true of all individual participants. Higher adaptation magnitude was observed for 40% of participants in cycle 2 versus cycle 1, and 45% had a higher adaptation magnitude in cycle 3 versus cycle 2. A Fisher's exact test showed no significant difference in the distribution of increasers and decreasers across cycles. Across the three cycles, 30% of participants decreased in adaptation magnitude each cycle and 10% increased, while 60% showed both an increase and a decrease.

Pearson correlations between adaptation magnitudes for individual participants were not statistically significant between any pair of hold phases (cycle 1 vs. cycle 2: *r*^2^ = 0.1, *p* = 0.18; cycle 1 vs. cycle 3: *r*^2^ = 0.03, *p* = 0.44; cycle 2 vs. cycle 3: *r*^2^ = 0.05, *p* = 0.33). Thus the observed decrease in group-average adaptation magnitude across cycles appears to be driven not by a systematic decrease in each participant's adaptation magnitude, but by an overall tendency for lower adaptation magnitude during a highly variable behavior.

### 3.4 Indicators of fatigue

During the final washout phase (trials 191–220), participants showed an unexpected change in pitch in the same direction as the feedback alteration, despite receiving unaltered feedback. To investigate whether participant fatigue at the end of the experiment could have caused this change in f0, two potential correlates of vocal fatigue, voice amplitude and f0 variability, were plotted over the course of the experiment.

**Figure 6:**
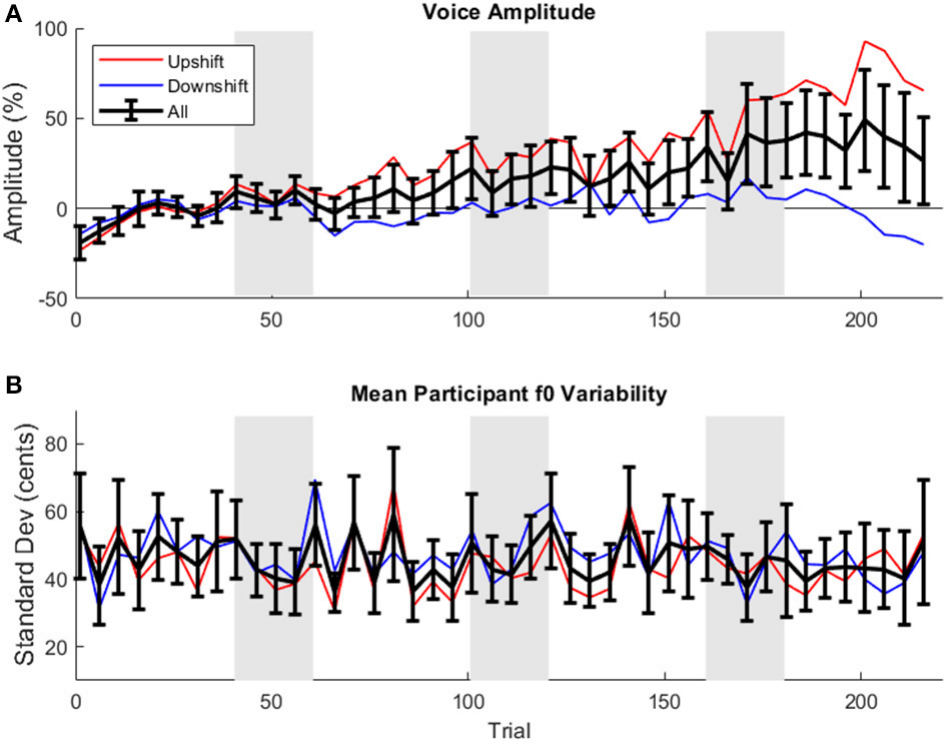
Possible indicators of fatigue. **(A)** Voice amplitude trended slightly louder over the course of the experiment. Participant responses differed between those who encountered upshifts and downshifts, with upshift participants (red) showing a greater trend toward louder vocal productions and downshift participants (blue) showing consistency in loudness until the last washout block, where productions trended slightly quieter. Plotted in black is the mean amplitude, normalized for each participant to the percent difference from the mean amplitude during the baseline period (trials 21–40), across participants in 5-trial blocks. Error bars indicate a 95% confidence interval. **(B)** Variability in f0 production remained consistent throughout the experiment and did not differ between participants who received upshifted (red) or downshifted (blue) feedback. Plotted in black is the mean across participants of standard deviations of 5 trial blocks. Error bars indicate a 95% confidence interval.

Voice amplitude was normalized for each participant to the percentage difference from that participant's amplitude during the first baseline period. Normalized amplitude was then averaged across all participants in 5-trial blocks. The top panel of Figure 6 shows that vocal amplitude overall tended to increase, rather than decrease, over the course of the experiment. Interestingly, there was a differential response between participants who received upshifted and downshifted f0 during the hold blocks, with downshift participants indeed showing a decrease in amplitude during the final washout phase, but upshift participants showing an increase in amplitude.

Next, variability in vocal pitch was calculated. Trial-by-trial f0 across the full task was normalized for each participant to cents based on the baseline period of the first cycle. For each participant, the standard deviation across each 5-trial block of the experiment was calculated, and then these standard deviations were averaged across all participants. The bottom panel of Figure 6 shows that mean f0 variability remained consistent throughout the experiment and was similar between upshift and downshift participants.

## 4 Discussion

This study is the first to observe adaptation behavior in a speaking task that features multiple cycles of identically pitch-shifted auditory feedback in a single session. The results replicate the previously observed adaptation response to a persistent shift in perceived f0 in an initial round of baseline, shift and washout (Jones and Munhall, 2000). They then show significant attenuation of adaptation magnitude in subsequent cycles of altered feedback. These results are consistent with our hypothesis that repeated cycles of f0 adaptation would be characterized by attenuated adaptation magnitude.

### 4.1 Explicit and implicit learning

In the arm movement domain, researchers have studied the mechanisms of learning through participants' responses to repeated cycles of adaptation. (Krakauer et al. 1999) showed that participants adapted faster to a second cycle of visuomotor adaptation administered the following day, in a phenomenon known as savings. (Kitago et al. 2013) found savings upon re-learning within a single session and found that this phenomenon depended on the type of un-learning between cycles, suggesting un-learning is not a passive forgetting but an active process. (Morehead et al. 2015) used participants' reports of intended aim to find that the measurement of savings was present in individuals' learning of conscious, reportable strategy (explicit learning), but not in the unconscious component of learning calculated by subtracting out the reported aim (implicit learning). In fact, several recent studies have shown that implicit learning processes show, rather than savings, an attenuation of adaptation magnitude upon re-learning (Avraham et al., 2021; Hamel et al., 2021; Leow et al., 2020). The attenuation, rather than savings, observed in the current study, supports the idea that f0 adaptation is driven mainly by implicit adaptation based on sensory prediction error. However, the possibility of some contribution of explicit learning cannot be ruled out with the design of the current study.

In recent follow-up studies of arm reaching, this attenuated re-learning has been attributed to anterograde inference, a phenomenon in which a prior learning task reduces learning in a later task. (Hamel et al. 2022) showed that the amount of interference was greater for reaching tasks that were more similar, while the amount of interference was similar for both identical and competing rotations on the same reaching task. This suggested that the inference was not a result of competing memories, but of temporarily reduced capacity for learning in the neural ensembles used to learn the first task. However, (Avraham and Ivry 2025) showed that in implicit adaptation, an extended baseline period could result in an attenuated adaptation magnitude, even when there was no previous learning period. This result suggests anterograde interference could be caused by a the competing memory of veridical feedback during the washout period. Future work may reconcile these results and test the extent to which they are applicable to the speech domain.

### 4.2 Previous studies of multiple adaptation in speech

Previous studies have presented multiple cycles of shifted auditory feedback in examining other questions, but none have compared identical f0 shifts in the same session. (Kapsner-Smith et al. 2024) measured f0 adaptation in separate sessions and found no significant difference in adaptation in the second cycle at the participant level. (Kim et al. 2025) measured identical cycles of F1 (not f0) adaptation in a single session and found no significant difference in adaptation in the second cycle at the participant level. In other studies, multiple cycles of pitch adaptation in the same session have featured slight but meaningful changes in task. (Hawco and Jones 2010) measured multiple cycles of f0 adaptation in a single session, but used a singing task where participants aimed for a different target pitch with each cycle, and found no difference in magnitude between cycles. (Alemi et al. 2020) performed two cycles of f0 adaptation in a single session but used different shift magnitudes in each cycle, so the response magnitude of the two cycles could not be compared. Although an attenuation effect was not measured in other studies featuring multiple blocks of adaptation in the domain of f0 control, each of these other studies contained a change in task or an extended time period between sessions, conditions which have been shown in the arm reaching domain to block anterograde interference (Hamel et al., 2021, 2022).

### 4.3 Test-retest reliability

The current study also provided an opportunity to examine the test-retest reliability of f0 adaptation in a single session. Previously, poor within-participant consistency has been reported in f0 adaptation for non-identical tasks and for identical tasks spaced across different sessions. (Kapsner-Smith et al. 2024) administered identical f0 adaptation tasks 3-6 weeks apart and found a low and nonsignificant intraclass correlation coefficient between the two sessions. In a study of two f0 adaptation tasks with different shift sizes, (Alemi et al. 2020) did find a significant correlation with their large sample size but found similarly weak correlation coefficients to those of the current study. Finally, (Dahl et al. 2024) found no significant correlation between participants' adaptation magnitude during a sentence task and a sustained vowel task. Similarly, the current study found no significant correlation between participants' adaptation magnitude in any pair of cycles. This supports previous findings by demonstrating that even for identical tasks in a single session, an individual's behavior in a single cycle of f0 adaptation does not necessarily predict that individual's behavior in a repeated presentation of the task.

In light of this finding, it is unsurprising that the group average decrease in response magnitude with each cycle of adaptation was not reflected in the behavior of individual participants. Only 30% of participants decreased in both the second and the third cycles, and more than half showed both an increase and a decrease in adaptation magnitude between cycles. The reduction in group average response magnitude should be interpreted as an overall tendency toward lower magnitude or more negative response in the group as a whole. Therefore, the study is limited in that it cannot predict re-adaptation behavior at the individual level.

### 4.4 Task-dependent adaptation magnitude

Adaptation magnitude in the first cycle, as measured by the fixed-effects coefficient of the posthoc linear mixed effects model, was 13.2 cents. This magnitude is smaller than many studies of f0 adaptation, however, observed adaptation magnitude has varied across studies and may depend on the task. For example, several studies involving singing tasks (in which participants were provided with a target pitch) have observed high magnitude f0 adaptation responses (Scheerer et al., 2016b, ~125 cents; Keough et al., 2013, ~50; cents Hawco and Jones, 2010, ~75 cents). Studies featuring a sustained vowel task in which no target pitch was provided have tended to have smaller adaptation magnitudes (Jones and Munhall, 2000, ~10 − 20 cents difference from no shift; Scheerer et al., 2016a, ~35 − 40 cents; Weerathunge et al., 2020, ~45 cents in the +5dB condition; Behroozmand and Sangtian, 2018, 62.3 cents in the short-term condition). The adaptation magnitude measured in the current study is in line with the smaller end of this distribution of speaking tasks, but also of note is that the current task used a single word prompt rather than a sustained vowel prompt. Few studies of f0 adaptation have used word utterance tasks, but a study involving both sentence and sustained vowel tasks (Dahl et al., 2024) found smaller f0 adaptation responses during utterance of words in a sentence than during a sustained vowel task, and smaller adaptation responses in non-emphasized words than in emphasized words in a sentence. Furthermore, the use of word utterances has been suggested as a possible explanation of increased frequency of following responses in f0 adaptation (Miller et al., 2023). This suggests that the magnitude of adaptation may depend on task, and that the current task involving single word utterances not emphasized within a sentence may expect smaller responses than previous studies using a sustained vowel task. Future work repeating the current study with a sustained vowel task may show a larger adaptation magnitude, however, the current study of word utterances may more closely resemble natural speech.

### 4.5 Repeatability of f0 adaptation

The attenuation of adaptation response after the first presentation cycle was much larger than expected, representing a 67% decrease in the second cycle and a 153% decrease in the third cycle from the initial adaptation. For comparison, (Avraham et al. 2021) observed a much smaller < 20% reduction in adaptation magnitude in the second cycle. A relatively small initial adaptation value, combined with a larger attenuation than expected, resulted in adaptation responses in the second and third cycles that were not significantly different from baseline in posthoc analyses of each cycle.

However, previous studies have shown that f0 adaptation is indeed repeatable in many conditions. (Kapsner-Smith et al. 2024) found significant f0 adaptation when procedures were repeated after 3-6 weeks. (Alemi et al. 2020) showed an adaptation response in a first and second cycle of adaptation separated by a non-adaptation task. (Hawco and Jones 2010) found no difference in repeated cycles of adaptation when each cycle featured a different target pitch, with an untimed break to rest in between. Thus the current study's finding of no significant adaptation after the first cycle may be specific to the continuous design of the current task, while an extended break or a change in task can result in a second significant adaptation.

### 4.6 Fatigue

We also considered whether the lack of adaptation in the second and third cycles could be caused by fatigue or boredom later in the task. Indeed, in a singing task, f0 adaptation magnitude was reduced in the dual-task condition, showing that attention has an impact on the magnitude of adaptation (Scheerer et al., 2016b). However, the total length of the task (220 trials, ~15 min) was shorter than other studies that have measured a significant adaptation response (Alemi et al., 2020; Behroozmand and Sangtian, 2018). In fact, (Behroozmand and Sangtian 2018) showed an adaptation response after a 100 trial baseline period, the same number of trials preceding the second hold phase in the current study. Thus boredom is unlikely to explain the absence of adaptation response after the first hold phase. Boredom was nevertheless mitigated as much as possible by the pseudo-randomized interleaving of trials containing a different prompt (“eat”) and by requiring user input to continue after each 20 trial block.

### 4.7 Washout

Finally, we considered whether the lack of significant adaptation after the first cycle was because participants had not fully returned to baseline when the next hold phase began. Since a washout phase twice as long as the hold phase was proportionally more washout than many studies who were able to measure adaptation a second time (Alemi et al., 2020; Avraham et al., 2021; Morehead et al., 2015; Kapsner-Smith et al., 2024), we do not believe this to be the case. Furthermore, no differences from baseline were found in early or late washout phases of cycle 1 or cycle 2, suggesting that baseline behavior was achieved during the washout period before each hold phase. Differences from baseline found in the washout period of cycle 3 were unexpected but were not followed by a hold phase. However, there may be multiple pathways in the formation of motor memories, not all of which can be measured by a return of behavior to baseline (Hadjiosif et al., 2023). Further studies are needed to understand these pathways in the context of speech motor control.

### 4.8 Decrease in f0 in final washout phase

Unexpectedly, we observed a large deviation from baseline in the final trials of the experiment (early and late washout of cycle 3), even though the auditory feedback was unaltered during this phase. This finding is not wholly unprecedented as (Weerathunge et al. 2020) also found a large increase in participants' f0 in one condition during a washout period after receiving upshifted f0.

One speculation is that this behavior may be related to a difference in fatigue between participants who received upshifted and downshifted feedback. Although across all participants, voices tended to become louder, not quieter, over time, participants who received different shift directions diverged in average amplitude. Those who received upshifted feedback became much louder at the end of the experiment while those who received downshifted feedback remained consistent for most of the experiment before trending quieter in the final six blocks. This may suggest a differential fatigue effect in which downshift participants became fatigued in the final trials of the experiment and consequently lowered their vocal pitch. Upshift participants, possibly less fatigued, drifted louder as they became accustomed to the loud feedback through the headphones and consequently raised their pitch. To pool participants who received different shift directions, the f0 responses of participants who received upshifted feedback were sign-inverted so that all opposing responses were shown as an increase in f0. This method amplifies changes in f0 related to the shift while reducing changes unrelated to the shift. While this was helpful for measuring the adaptation response, it may have also amplified a fatigue effect that differed by shift direction in the final trials of the experiment.

Regardless of the cause, the presence of this unexpected behavior does not affect the results of the study because it occurred during the final washout phase, and was not followed by any additional altered feedback.

### 4.9 Limitation

An additional limitation of the study is that a control condition with unaltered feedback was not tested. The use of randomized upshift and downshift conditions was used to mitigate any changes in pitch that were not caused by the feedback alterations, but future work would be strengthened by a no-shift condition.

### 4.10 Conclusion

Adaptation magnitude was significantly reduced in a second and third presentation of f0-shifted feedback compared with the first, which aligns with studies of implicit learning in limb movement tasks (Avraham et al., 2021; Hamel et al., 2021; Leow et al., 2020; Avraham and Ivry, 2025). On an individual level, speakers' adaptation magnitude in one cycle did not predict that speaker's behavior during another cycle, replicating in a single session what has been previously observed in separate sessions (Kapsner-Smith et al., 2024). These results support the characterization of f0 adaptation as an implicit learning phenomenon and can help inform computational models of f0 adaptation, which should account for the attenuation of response magnitude during re-learning and for high variability in individual behavior.

## Data Availability

The datasets presented in this study can be found in online repositories. The names of the repository/repositories and accession number(s) can be found below: https://github.com/jessicagaines/speech-analysis.
